# DEVELOPMENT AND EVALUATION OF COGNITIVE IMPAIRMENT SCREENING TOOL BASED ON EYE-TRACKING TECHNOLOGY

**DOI:** 10.1093/geroni/igad104.2745

**Published:** 2023-12-21

## Abstract

**Objectives:**

This study aimed to develop a cognitive impairment screening tool based on eye tracking technology (ET-CIS), and apply ET-CIS to community-dwelling older adults to evaluated its screening performance.

**Methods:**

ET-CIS were developed based on through systematic review, pre-experiments and expert consultation. We recruited older people in the community from July 2022 to November 2022 and collected data including demographics data, Montreal cognitive assessment, the mini-mental state examination, ET-CIS. The t-test and correlation analysis were conducted to screen out statistically significant parameters with of ET-CIS. The screening models were constructed using standardized score assignment, binary logistics regression analysis, and decision tree model in the training set, and were applied in the validation to evaluate the screening performance. The best model was selected as the final scoring model.

**Results:**

ET-CIS was constructed including applicable objects, pre-assessment preparation, screening dimensions, specific experiments and parameters. A total of 301 subjects were included, including 163 in the cognitively normal group and 138 in the cognitive impairment group. The results showed that the decision tree model results showed that the sensitivity of the training set was 0.752 and the sensitivity of the validation set was 0.818. We use the decision tree model as the final model.

**Conclusions:**

In this study, ET-CIS was developed including the evaluation of memory function, executive function, visuospatial function and abstract function. The screening model of ET-CIS in community-dwelling older people showed good discrimination, which demonstrated it could be used to effectively screen cognitive impairment in the community in the future.

